# Mr. Gibbon's Case of Ann Foulkes

**Published:** 1813-11

**Authors:** J. Gibbon

**Affiliations:** Bedford


*To the Editors of the Medical and Physical Journal.*

GENTLEMEN,

IN looking over your Journal for the present month, I find that Dr. Yeats reserves to himself the liberty of choosing the time and season for publishing the case of Ann Foulkes.

That the proper time for publishing her case was the period when his paper upon Ischuria made its appearance, will be apparent to every one, I humbly conceive, who peruses the following statement—that his conduct towards me and others in not doing so has been very extraordinary, and that we must necessarily feel hurt at his endeavours to reflect by implication upon our professional conduct, must be equally apparent.

Early in the spring of 1812, I repeatedly heard that there was a most wonderful case of disease in this town, and that the subject of it, A. F., evinced the most pious resignation under sufferings almost unparalleled.

I knew not whose patient she was, or had been. Motives of professional curiosity induced me to call upon her. I first did so on Thursday the 19th of March, and found her suffering apparent uneasiness from a tumefaction of the epigastric region. She informed me it was occasioned by a collection of urine, which was occasionally evacuated by vomiting. That every second or third day the accumulation was so great as to oblige her to take an emetic, which always enabled her to discharge the urine by the mouth very freely; but that if she did not take the emetic, her sufferings were much increased.

This state of things, she said, had existed about twenty weeks; and that during the whole of that time she had not voided a drop of urine by the natural passage.

Her general health did not appear to have suffered so much as might have been expected, from such an extraordinary, and, I may add, almost incredible disease. Indeed it struck me as being wonderful that she evinced so few symptoms of general illness, considering she had been so long confined to her bed.

This circumstance I wish to direct your attention to in a forcible manner. We all know how easy it is to feign numberless pains; but we as well know that long-continued visceral disease does universally show its effects in a direct and obvious manner.

Added to this, I have no hesitation in declaring that her digestion was at this time very healthy and perfect, from the well nourished state of the body, and from an inspection of well formed and healthy-colored fæces. It appeared to me very strange that these things could be so, whilst she complained of the dreadfully irritating effects of the discharged fluid upon the throat, fauces, and mouth. To satisfy myself upon this subject, I now thought it right to make every possible inquiry into the previous history of her diseases, and to pay great attention to these extraordinary symptoms. The more I did so, the more had I reason to fear there was some incorrectness in the relation of her story.

On the following day I called with a medical friend upon whose judgment I placed great reliance, and his astonishment was also excited.

We agreed to attend alternately at the periods of her taking the emetics. Previously, however, to doing this, we thought it essential to examine the state of the bladder by means of a catheter, and were *very much surprised* to find that no examination of this kind had been made in the early stage of the disease. To our great astonishment, both A. F. and her mother most obstinately refused to permit this simple operation. Our suspicions were then strongly excited, the more so, because they were very loud in their assertions of there being not a drop of urine in her bladder. They likewise affirmed that there had not been a drop of urine in that organ for several months. To strengthen this declaration, they assured us that Dr. Yeats, who had attended her very assiduously in the early period of her present illness, was of the same opinion. She was now attended solely by a druggist of this town, who, with the sanction and full approbation of Dr. Yeats, prescribed emetics, diuretics, &c. What steps were taken to assist in the ejection of the urine before Mr. Palgrave, the druggist, ordered emetics, I know not. Although it was represented that her sufferings were very dreadful at the time the urine first stopped, it was very extraordinary that her symptoms were not such as usually attend retention and suppression of the urine.

Upon a further and more accurate examination of the parietes parietes of the abdomen, I now discovered that the muscles of its lower part were always put into violent action, when the tumefaction of its upper part was most apparent, and in vain I requested that they might be relaxed. This, of course, led me to suspect, that the said tumefaction or inflation was in some measure dependent upon the action of these muscles, and I was certain that their action was principally voluntary. The truth of this suspicion was made very apparent some time afterwards; I happened to call at a period when she did not, or could not contract the muscles so forcibly, and the tumefaction had disappeared. Upon an examination of the epigastric region under these circumstances, I could discover no trace of visceral disease.

I had been assured the swelling always increased in proportion to the supposed accumulation of urine; but at the time I now allude to, she had not vomited urine, according to her own report, for some days, and I could only account for the absence of the swelling, by the quiet state of the muscles below. The hypogastric region was at this time fuller than usual, and I suppose she was incapable of practising her common habit of blowing out the epigastric region, owing to there being an unusually full state of the urinary bladder.

I had the less reason afterwards to be surprised at Dr. Yeats' believing that the tumefaction was now owing to a real collection of urine, because he did, in the most positive and unequivocal manner, declare his utter disbelief of the possibility of any person being able to cause such an inflation as I constantly witnessed, whereas nothing can be more easy.

There was no doubt of the nature of the fluid which the girl and mother positively asserted she (A. F.) vomited up. It was clearly urine. It now remained to be proved that such fluid was actually discharged by vomiting.

My friend went to her house on Saturday morning the 3d of April; her mother said she had just swallowed the emetic, and that it was beginning to operate. He staid a considerable time, during which she appeared to be very sick and retched often, but neither urine, nor any thing of an urinous smell was brought up. With much persuasion he induced her to swallow some warm water; this returned by vomiting, but quite free from any admixture of urine. He then left her, perfectly convinced of the incorrectness of the whole story, as he was before assured of its utter improbability.

A few days after I went to the house, whilst she was said to be under the operation of an emetic. When I entered the chamber, her mother shewed me about half a pint of fresh, transparent, and apparently healthy urine, free from the the least admixture of mucus or any other fluid or solid matter, which she informed me her daughter had just vomited up. On looking at it, I felt quite sure it could never have been discharged from the stomach or throat of a person under the influence of an emetic, because of the total absence of that matter, which must necessarily be in the stomach, throat, or mouth, of every living person. This quantity was, however, very small, compared to that which I had been informed always came up when an emetic was taken; I therefore waited patiently for more urine to make its appearance—she professed to be very sick, but none came.

Her mother then told me it was not a vomiting day, and that some days she did not vomit up the urine so freely as she did on others; contrary to the story they first told me, when it was not supposed I should be sufficiently inquisitive, to insist upon seeing this urinous matter vomited.

At this time the mother likewise expressed a hope that I was satisfied what she had showed me was urine. I certainly was, but equally so, that it had been discharged from the body in the natural manner.

To put the matter to rest, I now spoke in a very decided manner, about the propriety of allowing a catheter to be used. I pledged myself that she would be cured by it, but no persuasion was of any avail. I afterwards called at the house, and expressed such displeasure at their continuing to refuse the assistance which I offered to procure, as made them endeavor to excuse themselves under the cloak of religion.

Upon offering to send for Dr. Yeats, or any other medical gentleman they might prefer, in order to make the examination with the catheter, they now told me it would be of no use, because they were both determined not to submit to it—being quite sure it was wrong to try to take the work out of the hands of God, who had thought fit to afflict her in this very extraordinary manner: and both A. F. and her mother declared they thought it the duty of the former to wait God's time for bringing about a cure, if it was ever to take place. They appealed to me upon the harshness of my conduct, in appearing to disbelieve a person, who was so generally respected for the piety of her character; and said, that I ought to be ashamed of myself, (or words to that effect,) for withholding my assent to those reports which Dr. Yeats, and many other persons, implicitly believed, upon their simple assertion. This determined me to inform her charitable supporters, and the overseers of her parish, of my doubts respecting the veracity of her story;—and I hinted to them what was my determination, in case their free consent was not soon given, to the proposal above stated. I was, however, prevented from doing so, by receiving information from the mother of A. F. of a great change in her daughter's complaints. She was said to be seized with symptoms of common fever, which was accompanied with apparent stupor, of two or three days duration, and at the close of this symptom, the urine found its way from the stomach to its proper organ, from which it has been regularly discharged to this time. I once saw her during this declared stupor, which I considered as deceptious, from the absence of those symptoms which are its usual concomitants; neither was any change or derangement of the urinary organs apparent.

Before the asserted return of the urine to its natural channel, I felt fully assured that a proper examination of the bladder would prove the total falsity of A. F.'s tale, and upon that account I thought it my duty to insist upon its being made. Probably the parties concerned well knew the same. Nay, I add, it is very unlikely that a case so painful as retention or suppression of urine has ever existed, in which the sufferer objected to the proper methods of attempting to give relief.

I must now touch upon some circumstances connected with this second Tutbury tale.

It has been objected to my opinion of the case being a clear attempt at imposture, that there was no adequate motive. I am not bound to prove any motive, but I can venture to assert, that it would astonish most persons at a distance from Bedford, to learn what was the greatest quantity of provision, clothing, and money, sent to A. F.'s house in one month, previously to my declaring this opinion publicly.

But for the circumstance of Dr. Yeats being much implicated in the foregoing statement, I should have sent you the result of my inquiries into this reputed state of Ischuria long ago. With the greatest reluctance to become personal, I feel obliged, in justification of my own character, and the opinions of other medical men, to make some observations on the papers which have appeared in your Journals since April last. As Dr. Yeats all along did, and I suppose to this moment does believe, that this marvellous vomiting actually took place, he, of course, felt an obligation to controvert the opinions which I and other medical men gave upon the subject, namely, that the declarations of Ann Foulkes and her mother were untrue, and utterly impossible.

I, and many others, distinctly understood from Dr. Yeats, that he intended (or pledged himself) to prove both the truth and and possibility of A. F.'s story. The abstract question of vomiting urine for a length of time was never thought of, unconnected with the peculiarities of the case in question. Is it not then very extraordinary, that Dr. Yeats should try to reflect upon our opinions, by implication, in a manner which is unfair? But for the occurrence of this case, his paper upon Ischuria would never have been written. When I first saw A. F., and during the whole time I visited her, previously to the asserted return of the urine to the bladder, she was attended by Mr. Palgrave, a druggist, of this town. It is strange to tell, that Dr. Yeats gave up the care of this interesting case to him alone. Mr. P., under the controul of Dr. Yeats, gave the emetics and gave the diuretics. Without calling in any of the medical men of Bedford to assist him in investigating this wonderful story, Dr. Y. rested satisfied that all he heard was true.

When my mind was decidedly made up on this subject, and whilst I considered A. F. as being more immediately under the care of the druggist, I told Dr. Yeats, in a truly friendly manner, that I was in possession of such facts as compelled me to consider her in the light of an impostor. He then seemed to think that my opinions were not founded upon facts. He assured me, that he paid so much deference to the assertions of the parties interested in this (as I supposed deceptious) affair, that he could not think of changing his opinions respecting her case. In addition to the facts which he thought would satisfactorily prove the truth of all this marvellous story, he is now in possession of the oaths of two persons who were in the habit of waiting upon or nursing A. F., which oaths I may hereafter show are to be regarded in a very suspicious point of view.

Not only did I inform Dr. Y. of my opinion, but I sent him a written statement, containing the substance of the foregoing history. He, in return, was kind enough to favor me with the perusal of a very long account of her case, which I understood was intended for publication; and, if I am not misinformed, has been already presented to a medical society for publication. If I had wanted any information to strengthen my previous opinion, or clear up any remaining doubts, the inspection of Dr. Y.'s case-book would have been amply sufficient; it corroborated, in a remarkable manner, what A. F. had previously told me about the absence of symptoms, which a better informed impostor would have spoken of when mentioning what took place at the time she said the urine first stopped.

The case now appeared so clear to me, and all those medical men with whom I had any intercourse or correspondence, ence, that I could not think it probable Dr. Y. would venture to commit himself by writing upon the subject, except it was to acknowledge that he might have been imposed upon: still less, after what had passed, could I suppose it possible he would write upon Ischuria, or vomiting of urine, without giving the particulars of this case.

It must be evident to every one, that if I say A. F. is an impostor, because of the utter impossibility of her having vomited urine in the manner reported above, I have a right to expect that the man who controverts my opinion will do it in a fair and candid manner.

It was unpleasant enough for me, that in the simple discharge of my duty, I was obliged to incur the ill opinion of many well-meaning persons, whose good opinion I have been always solicitous to deserve; but that Dr. Y. could go such a round-about way to work to try to make a fact of what I suppose a fable, astonishes me much indeed. I have to assure you, that it was principally out of tenderness for his professional character, that I abstained from publishing my opinions of this case before; and I now feel justified in calling upon him, through the medium of your Journal, to give his history of the whole case, or acknowledge that he was imposed upon.

Dr. Y. might have chosen the time and season for publishing upon Ischuria; but I must repeat, that if I did not call upon him to publish this case, according to his promise, I cannot willingly submit to his assertions in your Journal for the present month. The point in dispute between him and me, and I believe between him and other medical gentlemen, is, simply, whether it was possible for Ann Foulkes to have vomited urine in the manner, and for the length of time, she declares she did. I do not act more unfairly towards Dr. Y. in relating the following story, than he has done towards others and myself, in writing his paper upon Ischuria, without giving the case of A. F. in connection with it.

A woman, who lived in Essex a few years ago, declared, as did her friends, that she was in the habit of sweating a vast quantity of living tadpoles, or animalculæ, of a visible size, which were seen very merry in the buckets full of fluid that was said to have exuded from her skin. This excited the wonder and curiosity of the neighbourhood. When it was understood that a medical gentleman believed it, many persons went to see this phenomenon from a distance, and made liberal contributions towards defraying the expence of washing and drying the great number of cloths that were used to absorb this unusual discharge from the skin, and to furnish her with wine to keep up her strength. At last, a physician of great respectability insisted upon seeing these animals make their way through the skin. That could not be acceded to. I scarce need add, that, upon further inquiry, it was proved, the persons in connection with this woman supplied her with tadpoles from a neighbouring ditch. I am, Gentlemen,

Your obedient Servant,

*Bedford, Sept.* 9, 1813. J. GIBBON.

